# Adenosine A1 Receptor Agonist (R-PIA) before Pilocarpine Modulates Pro- and Anti-Apoptotic Factors in an Animal Model of Epilepsy

**DOI:** 10.3390/ph14040376

**Published:** 2021-04-18

**Authors:** Daniele Suzete Persike, Rebeca Padrão Amorim Puccinelli, Maria José da Silva Fernandes

**Affiliations:** 1 Departamento de Neurologia/Neurocirurgia, Disciplina Neurociência, Escola Paulista de Medicina, Universidade Federal de São Paulo-UNIFESP, Rua Pedro de Toledo, 669, CEP, São Paulo 04039-032, Brazil; daniele_persike@protonmail.com (D.S.P.); becapadrao@gmail.com (R.P.A.P.); 2 Department of Medicinal Chemistry, College of Pharmacy, University of Dohuk-UoD, Kurdistan Governmental Region, Dohuk 1006AJ, Iraq

**Keywords:** epilepsy, pilocarpine, adenosine, A1-receptor, neuroprotection, caspases, cathepsin D, AKT, HSP-70

## Abstract

We aimed to characterize the mechanisms involved in neuroprotection by R-PIA administered before pilocarpine-induced seizures. Caspase-1 and caspase-3 activities were assayed using fluorimetry, and cathepsin D, HSP-70, and AKT expression levels were assayed using Western Blot of hippocampal samples. R-PIA was injected before pilocarpine (PILO), and four groups were studied at 1 h 30 min and 7 days following initiation of status epilepticus (SE): PILO, R-PIA+PILO, SALINE, and R-PIA+SALINE. At 1 h 30 min, significantly higher activities of caspase-1 and -3 were observed in the PILO group than in the SALINE group. Caspase-1 and -3 activities were higher in the R-PIA+PILO group than in the PILO group. At 7 days following SE, caspase-1 and -3 activities were higher than in the initial post-seizure phase compared to the SALINE group. The pretreatment of rats receiving PILO significantly reduced caspase activities compared to the PILO group. Expression of HSP-70, AKT, and cathepsin D was significantly higher in the PILO group than in the SALINE. In the R-PIA+PILO group, the expression of AKT and HSP-70 was greater than in rats receiving only PILO, while cathepsin D presented decreased expression. Pretreatment with R-PIA in PILO-injected rats strongly inhibited caspase-1 and caspase-3 activities and cathepsin D expression. It also increased expression levels of the neuroprotective proteins HSP-70 and AKT, suggesting an important role in modulating the cellular survival cascade.

## 1. Introduction

Epilepsy is a chronic neurologic disease that affects about 65 million people worldwide, representing a substantial proportion of patients in neurological clinics and a substantial global disease burden [1]. About 40% of patients present with temporal lobe epilepsy. Despite the growing number of antiepileptic drugs commercially available, about 30% of patients have refractory epilepsy. Hippocampal sclerosis is the most common histopathology observed in patients with mesial temporal lobe epilepsy (MTLE) [2]. Mesial temporal sclerosis (MTS) is a term used to refer to significant pathological changes involving not only the hippocampus but also the amygdala and entorhinal cortex. A task force of the International League Against Epilepsy (ILAE), based on the cell loss patterns, classified MTS into MTS type 1 (neuronal loss mainly in CA1 and CA4), MTS type 2 (CA1 sclerosis), MTS type 3 (end folium sclerosis), and no MTS. In specimens classified as no MTS, only intense reactive gliosis is observed without neuron cell loss, suggesting a pivotal role of the glia in the seizure generation [3]. MTS is always associated with granular cell dispersion in the dentate gyrus related to cognitive deficit [4,5]. Many lines of evidence point to glia-mediated inflammation and excitation related to seizure triggering, but this complex interaction needs elucidation.

Experimental models of epilepsy help describe cellular and molecular changes that occur during epileptogenesis and ictogenesis. Pilocarpine-induced epilepsy in mice or rats has been widely used as an experimental model of MTLE because it reproduces the primary electroencephalographic, histopathological, and behavioral characteristics of the disease [5,6,7,8]. High-dose pilocarpine induces status epilepticus (SE). Only 2–5 h of SE duration is sufficient to induce subsequent stages of epileptogenesis, i.e., the latent period (7–14 days duration, 7 days average) with normal behavioral and EEG, and the chronic period, characterized by spontaneous and recurrent seizures, persisting throughout the life of the animal [6,7,8,9]. Cognitive deficit is one of the behavior changes observed following cell death caused by seizures [10].

Glial activation with the consequent release of cytokines, chemokines, and costimulatory molecules is the leading player involved in epileptogenesis and ictogenesis following SE. Seizures can activate apoptotic pathways [11]. Otherwise, seizures can occur following caspase activation [11,12,13]. The inhibition of caspase-1, -3, -8, and -9 can promote neuroprotection following seizures [14,15] and represent a good strategy for preventing cell death during epileptogenesis.

Besides, studies show that the so-called “apoptotic caspases” are more than just killers [16,17,18]. In the CNS, these enzymes, particularly caspase, which is the most abundant cysteine protease, can contribute to axon guidance, synaptic plasticity, and neuroprotection [15]. Besides, the apoptotic executioners’ caspases 3, 7 and 8, can promote microglial activation in the absence of cell death. It was understood to be part of the innate immune response to chronic neurodegenerative diseases [16]. Thus, caspase activation can be part of adaptive cellular responses under a delicate balance to determine normal cellular functioning or a cell death activation. In this adaptative response, activated caspase may induce upregulation of cytoprotective proteins like heat shock proteins (HSPs), limiting the caspase cleavage [19,20].

HSPs are constitutive and inducible chaperone molecules involved with the maintenance of cellular protein homeostasis as it prevents protein misfolding and aggregation and inhibiting caspase cascade and apoptosis during injury [19,20]. HSP 70 has been extensively studied in epileptic conditions. A high level of HSP has been reported in hippocampal specimens from patients with TLE [21] and in an experimental model of epilepsy [22], suggesting neuroprotection. However, an increased level of HSP70 following seizures induced by kainic acid was considered only a marker of stressed neurons by some authors [23]. In addition to HSP, the anti-apoptotic protein kinase B (AKT) is upregulated in resected TLE tissue, and AKT is a crucial survival factor following brain injury [24]. Activation of AKT by mammalian target of rapamycin (mTOR) signaling pathway (mTORC2) can prevent cell death caused by seizures [25,26].

Cathepsins are proteases that can be upregulated under neuroinflammation and reactive glial cell conditions [27,28]. Cathepsin D is the major endolysosomal aspartic protease. This enzyme has essential physiological functions as myelinization during the development [29]. However, it can be involved in the pathogenesis of several neurodegenerative diseases like Alzheimer’s disease [28,30], Huntington’s disease [31], Parkinson’s disease [32], age-related cell death [33,34] and epilepsy [35]. Activated microglia in the brain is the primary source of cathepsins during aging [28] and is also the principal pathophysiological finding of numerous neurodegenerative disorders, including TLE models [35,36]. Although neuroinflammation is one of the main pathophysiological findings of TLE, the role of cathepsins in neurodegeneration resulting from seizures needs more clarification.

Status epilepticus can cause an excitotoxic level of glutamate release, overstimulation of glutamate receptors leading to enhanced intracellular calcium level that activates proteolytic enzymes leading to cell death epileptogenesis [37]. Thus, it is of great clinical interest to inhibit calcium influx to prevent cell loss and epileptogenesis.

**Figure 1:**
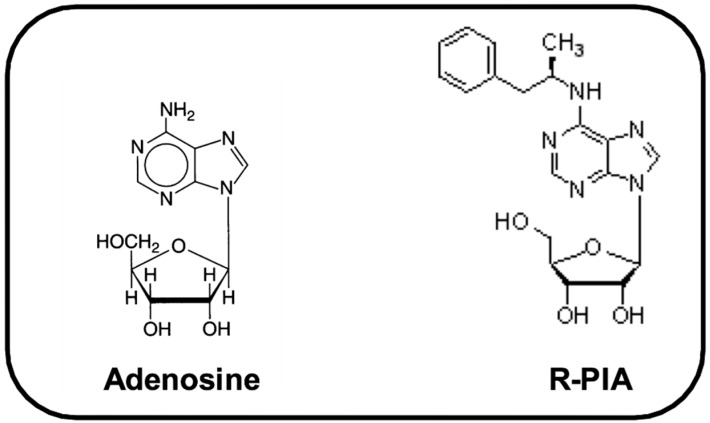
Molecular structure of compounds adenosine: (2*R*, 3*R*, 4*S*, 5*R*)-2-(6-amino-9*H*-purin-9-yl) 5-(hydroxymethyl)oxolane-3,4-diol, and its A1 agonist R-PIA: [R-N6-(2-phenylisopropyladenosine)].

Adenosine is an endogenous neuromodulator released in the brain during seizures, ischemia, and hypoxia [38,39,40] and can inhibit or stimulate synaptic transmission by activating A1 or A2a receptors, respectively [41]. In previous studies, we showed that pretreatment with the selective A1 receptor agonist R-PIA (Figure 1) had antiepileptic and neuroprotective effects in rats subjected to pilocarpine [42,43,44]. R-PIA may induce neuroprotection by activating pre-synaptic A1 receptors that attenuate calcium influx through voltage-dependent calcium channels (mainly N-type), decreasing glutamate release [45,46,47,48]. We also showed that R-PIA-induced neuroprotection in the pilocarpine model was assigned to a reduced imbalance between local cerebral blood flow and local consumption of glucose in brain areas associated with onset and propagation of seizures [44]. Little is known about the cellular mechanisms activated by A1 receptor stimulation using R-PIA. Therefore, in the present study, we studied the role of R-PIA administered before pilocarpine on the modulation of pro-apoptotic factors (caspase-1, caspase-3, and cathepsin D) and anti-apoptotic markers (AKT and HSP-70) in the hippocampus.

## 2. Results

The non-parametric Scheirer–Ray–Hare test was used to analyze the data of caspase-1 and -3 activities and showed the significant effect of groups (*p* < 0.0001) but not effect of time (*p* > 0.13). Comparisons between the groups were made by the Kruskal–Wallis test and post hoc Wilcoxon and the data are described below.

### 2.1. Caspase-1

**Figure 2:**
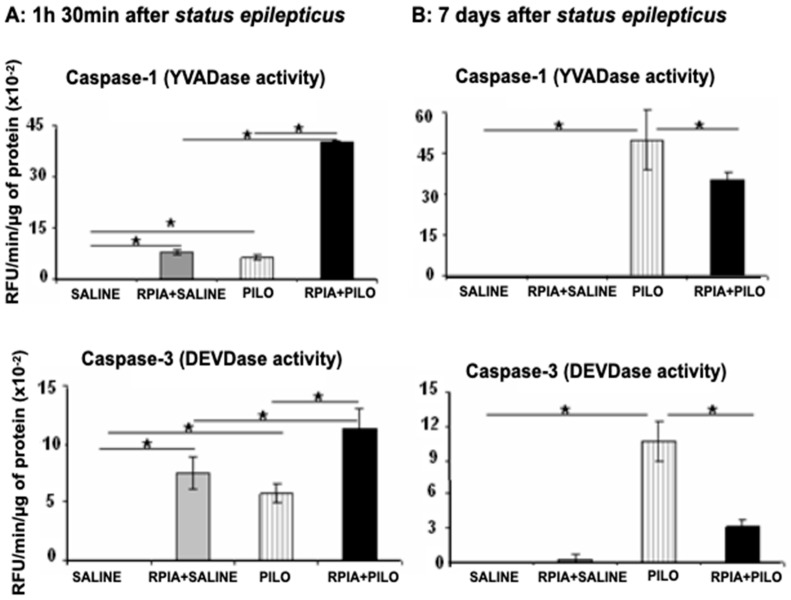
Fluorometric assay of caspases in the hippocampal homogenates from rats studied at various time points following pilocarpine-induced status epilepticus. R-PIA was injected 15 min before saline (R-PIA+SALINE) or pilocarpine injection (R-PIA+PILO). Significant increases in caspase-1 and -3 activity were observed in the R-PIA+SALINE, PILO, and R-PIA+PILO groups compared to the SALINE group. The activity of caspase-1 and-3 was higher in the R-PIA+PILO group than in the PILO group (**A**). Seven days following the SE onset (**B**), there was a significant increase in the activity of caspase-1 and-3 in the PILO and R-PIA+PILO groups compared to the control group. However, in the R-PIA pretreated rats, there was a significant reduction in the activity of caspases-1 and -3 when compared to the PILO group. Bars represent the mean ± SD of fluorescence measurement (RFU/min/µg of protein) of four independent experiments (*N* = 5) made in duplicate. * *p* < 0.01, Kruskal–Wallis and post hoc Wilcoxon tests.

At 1 h 30 min following SE onset, there was significantly higher caspase-1 activity in the PILO and R-PIA+PILO groups than in the R-PIA+SALINE or SALINE groups (Kruskal–Wallis, *p* < 0.001; Figure 2A). Caspase-1 activity was higher (+60% and +80%) in the PILO and R-PIA+SALINE groups, respectively, than in the SALINE group (*p* < 0.008). The activity of caspase-1 was greater (+540%, *p* < 0.008) in the R-PIA+PILO group than in the PILO group. Seven days following the SE induction, the activity of caspase-1 was significantly higher in the PILO-treated rats than in the SALINE and R-PIA+PILO groups (Kruskal–Wallis, *p* < 0.001; Figure 2B). Caspase-1 had less activity in the R-PIA+PILO group than in the PILO group (−29%, *p* < 0.008) (Figure 2A,B).

### 2.2. Caspase-3

At 1 h 30 min following SE, caspase-3 activity was significantly higher in the PILO, R-PIA+PILO, and R-PIA+SALINE groups (Kruskal–Wallis, *p* < 0.001). Caspase-3 activity was higher (+56% and +72%) in the PILO and R-PIA+SALINE groups, respectively, than in the SALINE group (*p* = 0.01; Figure 2A). In the R-PIA+PILO group, caspase-3 activity was higher than in the PILO group (79%, *p* = 0.01). Seven days following SE, caspase-3 activity was higher only in the PILO group (Kruskal–Wallis, *p* < 0.001) and was 71% higher than the R-PIA+PILO group and 108% higher than that of the SALINE group (*p* = 0.01) (Figure 2B). The caspase-3 activity measured in the PILO group at seven days was higher (+46.8%, *p* = 0.01) than that analyzed at the initial phase following SE (Figure 2A,B). No activity was observed in the SALINE and R-PIA+SALINE groups (Figure 2B).

### 2.3. AKT, HSP 70, and Cathepsin D Expression

**Figure 3:**
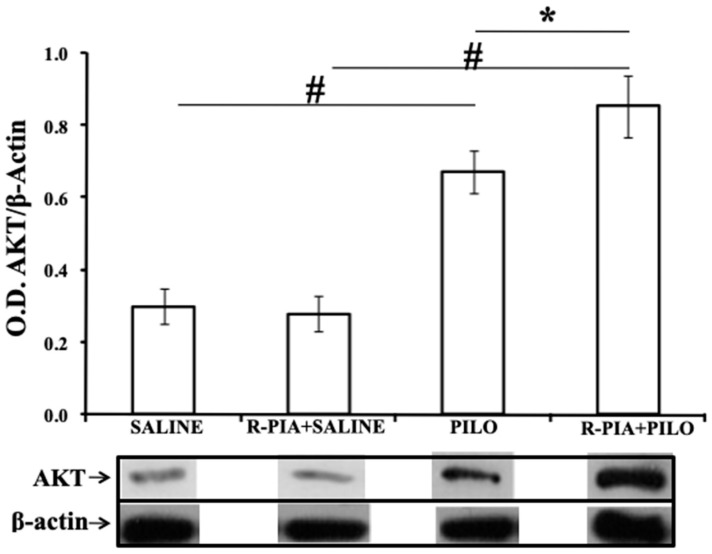
Expression of AKT (protein kinase B) in hippocampal samples of rats from SALINE, R-PIA+SALINE, PILO, and R-PIA+PILO groups. Bars represent the mean ± standard deviation of optical density for each group (N = 4/group), normalized to β-actin. (R-PIA+PILO vs. PILO, * t*P* = 0.05; R-PIA+PILO vs. R-PIA+SALINA, PILO vs SALINA, **^#^** *p* = 0.03; Kruskal–Wallis with Wilcoxon post hoc).

**Figure 4:**
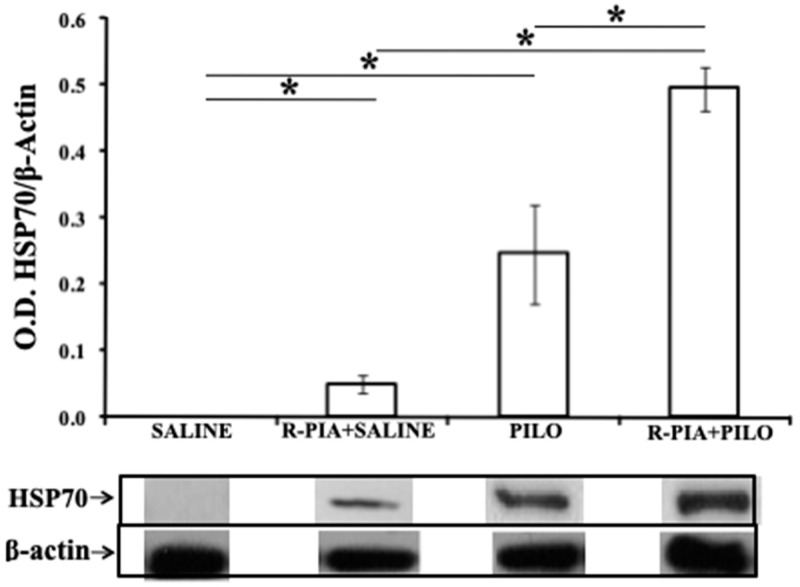
Expression of HSP70 in the hippocampal samples of rats from all studied groups. Bars represent the mean ± standard deviation of optical density for each group (*N* = 4/group), normalized to β-actin levels. * *p* = 0.03 (Kruskal–Wallis with Wilcoxon post hoc).

**Figure 5:**
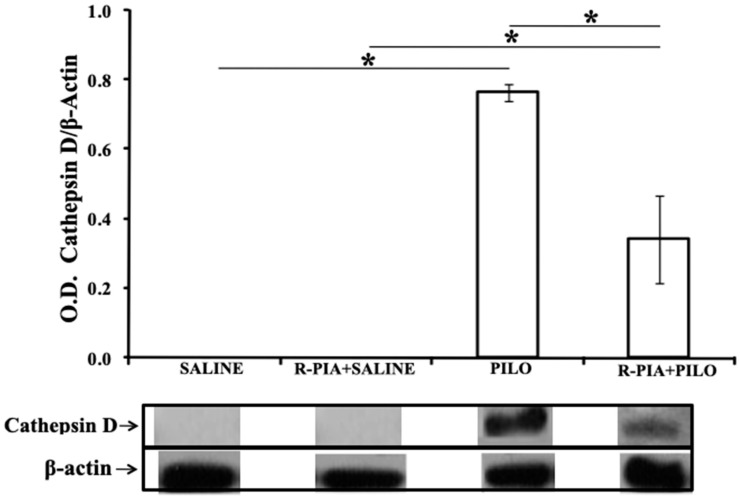
Expression of cathepsin D in the hippocampal samples of rats from all studied groups. Bars represent the mean ± standard deviation of optical density for each group (*N* = 4/group), normalized to β-actin levels. * *p* < 0.03 (Wilcoxon).

The expression of AKT, HSP 70, and cathepsin D proteins was studied using Western Blot, and differential expression of those were seen between the groups (Kruskal–Wallis, *p* = 0.003 for AKT and *p* = 0.002 for HSP-70 and cathepsin D). There were significantly higher expression levels of AKT in the PILO group than in the control SALINE or R-PIA+SALINE groups (*p* = 0.03), and a similar level than R-PIA+PILO (*p* = 0.06; Figure 3). There were also significantly higher expression levels of HSP-70 in the R-PIA+SALINE, PILO, and R-PIA+PILO groups than in the SALINE group (Figure 4). However, higher expression of HSP-70 was observed in the hippocampus of rats pretreated with R-PIA receiving PILO than in rats receiving only PILO (*p* = 0.03; Figure 4). Higher expression levels of cathepsin D were observed in the PILO group than in the SALINE or R-PIA+SALINE groups (*p* = 0.03; Figure 5). A 50% reduction in the cathepsin D expression was observed in the hippocampus of the R-PIA+PILO group compared to the PILO group (*p* = 0.03; Figure 5). No expression of cathepsin D was observed in the control groups (SALINE and R-PIA+SALINE).

## 3. Discussion

We measured the activity levels of the pro-inflammatory caspase-1 and the apoptotic enzyme caspase-3 in the hippocampus of rats at various time points following pilocarpine-induced SE (1 h 30 min and 7 days). We also measured levels of cell markers involved with neuroprotection (AKT and HSP-70) and cell death (cathepsin D) to characterize the molecular cascade activated by seizures in the hippocampus during these periods.

### 3.1. Caspases and Cathepsin D Activation Following Pilocarpine-Induced SE

We found increases in the activities of caspase-1 and -3 in the hippocampus of rats from the PILO group following 1 h 30 min of SE. Pretreatment with R-PIA to rats receiving pilocarpine increased the activity of caspases compared to activity levels in rats treated only with PILO. At 1 h 30 min following saline treatment, caspases-1 and -3 were also significantly increased in rats pretreated with R-PIA, suggesting a role of R-PIA in caspase activation.

At seven days following SE, no caspase activities were observed in any control (SALINE and R-PIA+SALINE), although caspase-1 and -3 activities remained very high in the PILO group; however, there were reduced activities in the R-PIA+PILO group. These findings suggest that R-PIA administered before pilocarpine activates caspases-1 and -3 as early as 1 h 30 min following SE onset and inhibits apoptotic cascades and inflammation resulting from seizures 7 days later. Caspases are serine-proteases involved with apoptosis. Three groups of caspases have been described: those related to inflammation (caspases-1, -4, -5, -11, and -12), apoptotic executioners (caspases-3, -6, and -7), and initiators of apoptosis (caspases -8, -9, and -10). There is evidence to suggest that SE activates caspase-dependent and independent excitotoxic mechanisms, as well as p53, Bclx, and endonucleases leading to apoptosis [49]. Our previous study showed that pilocarpine-induced SE activated inflammatory caspase-1 and apoptotic executioner caspase-3 as early as 1 h 30 min following SE onset during epileptogenesis caused by pilocarpine [50]. Otherwise, seizures can occur following activation of caspase-1 [51], caspase-3 [12,52,53], caspase-8 [54], or caspases-2 and -9 [13]. These findings suggest that blockade of caspases-1 and -3 may be beneficial following SE.

Our findings suggest that R-PIA increased caspase activity in the initial phase of SE, and significantly reduced it 7 days later, a time in which expressive neuroprotection was observed in the hippocampus [43,44].

Inflammatory mediators and cytokines are rapidly synthesized during seizures, and some of them have a pro-convulsant activity [14]. Previous studies have shown that interleukin-1β and microglia are increased during seizures, and intracerebral injection of IL-1β antagonist has a powerful anticonvulsant effect [55,56]. In agreement with Ravizza and coworkers [14], we suggest that caspase-1 activation exacerbates seizures and cell death in the pilocarpine model. Many authors have identified caspase inhibitors as neuroprotectors and anticonvulsant strategies for epilepsy [11,12,14,53]. Although caspase-1 and caspase-3 were activated early (1 h 30 min) in saline control rats, no changes were observed 7 days later. These data suggest that R-PIA can activate caspases-1 and -3 in the hippocampus without causing observable neuropathology. Our data contrast with those of Zhai et al. (2016), who found suppression in the level of caspase-3 following intracerebral hemorrhage in rats [57]. Further study is needed to clarify this concept.

Cathepsins are lysosomal cysteine proteases that are involved in physiologic processes such as proenzyme activation, antigen presentation, tissue remodeling, bone matrix resorption, and pathologic processes such as facilitation of tumor invasion and modulation of programmed cell death [58]. However, lysosomal disruption leading to cytosolic cathepsins release may trigger apoptosis [59]. Cathepsin D participates in the mechanism of autophagy, a cellular process undertaken by neurons in the central nervous system to transport unneeded constituents to lysosomes [60]. This process is essential for the maintenance of cellular metabolism under physiological conditions. Hypoxic/ischemic brain injury at birth (a significant cause of epilepsy, cerebral palsy, and mental retardation) causes energy failure, oxidative stress, and unbalanced ion fluxes, leading to robust autophagy in neurons of the brain [60]. Acceleration of autophagy induced by the loss of lysosomal proteinases such as cathepsins D, B, and L, or hypoxic/ischemic brain injury, causes neurodegeneration [60].

We also measured expression levels of cathepsin D to analyze the participation of lysosomal proteases in the mechanism of cell death caused by epilepsy induced by pilocarpine. We found concomitant upregulation of cathepsin D and caspases in the hippocampus of rats in the latent phase of the pilocarpine model, suggesting that various cell death mechanisms are activated during SE to neurodegeneration in the pilocarpine model. Inhibition of cathepsin D and caspases-1 and -3 may represent a promising neuroprotective strategy during SE.

### 3.2. Neuroprotection by R-PIA

Our previous study showed that R-PIA has a neuroprotective effect in the PILO model; pretreatment reduced the mortality rate, increased the latency to the onset of SE, decreased the number of animals developing SE, and decreased neurodegeneration [43]. In the present study, we showed that expression levels of AKT and HSP-70 were significantly elevated in the hippocampus 7 days following SE; however, these increases were more significant in animals pretreated with R-PIA before PILO, suggesting a potentiation of these expressions by R-PIA.

Corroborating our data, Gervitz et al. showed that levels of phosphorylated AK/PKB were elevated under in vivo ischemic conditions and an A1 receptor-dependent manner [61]. In an in vitro study, these authors demonstrated high levels of AKT expression in hippocampal slices treated with an adenosine A1 agonist. The authors concluded that A1 receptor activation, either by endogenous adenosine (in vivo) or by an adenosine agonist (in vitro), stimulated survival pathways.

Several authors showed that high-affinity adenosine A1 receptors are expressed in high density in the hippocampus and that these receptors are responsible for the tonic inhibition of the hippocampus [62,63]. Using knockout mice for the A1 receptor, Fedele et al. [64] showed that these receptors are essential for preventing the spread of seizures induced by kainic acid.

**Figure 6:**
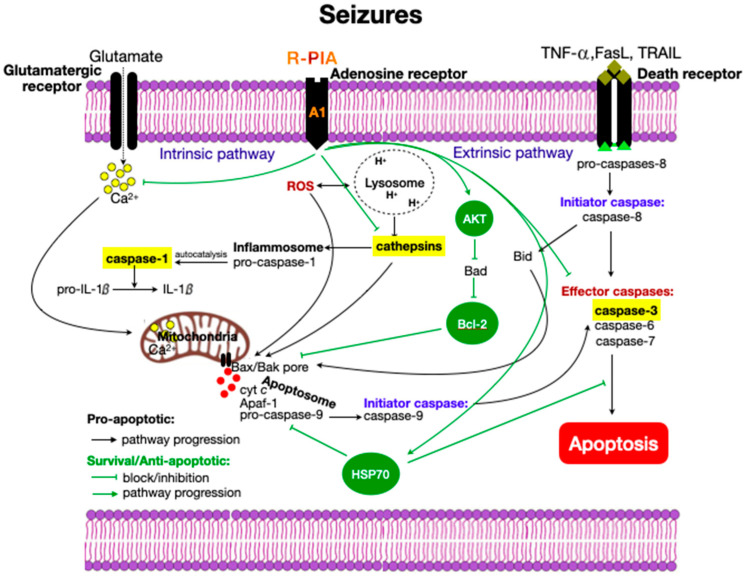
Molecular signaling activated by seizures leading to cell death by apoptosis. Two pathways, extrinsic and intrinsic, may trigger apoptosis. TNF (tumor necrosis factor) activates cell-surface death receptors in the extrinsic pathway, leading to an intracellular death-inducing signaling complex containing Fas-associated death domain and caspase-8 or -10. A caspase cascade is initiated by the activation of “effector” caspases, such as caspase-3, which cleave essential intracellular structural and survival proteins and lead to apoptosis. The intrinsic pathway is triggered by increased intracellular calcium, free radicals, and dimerization interactions of Bcl-2 family proteins that regulate mitochondrial dysfunction. Mitochondrial alteration leads to cytochrome c release, which activates the apoptosome formed by Apaf-1 (apoptotic protease-activating factor 1) and caspase-9, followed by downstream executioner caspases. Reactive oxygen species (ROS) and intracellular calcium can alternatively induce lysosomal disruption, releasing cathepsins. Cathepsins may directly trigger cytochrome c release, leading to caspases activation. Caspase-1 is activated by the inflammasome and converts pro-IL-1B to mature IL-1B. These pathways can be modulated by survival proteins AKT, anti-apoptotic Bcl-2, and HSP-70. The A1Rs (adenosine A1 receptors) are effective modulators of excitatory transmission where they exert combined pre-, post-, and non-synaptic effects to decrease glutamate excitability. In this manner, the activation of presynaptic A1R by R-PIA reduces the influx of calcium through voltage-sensitive calcium channels and consequently inhibits the release of glutamate; at the post-synaptic level, it inhibits voltage-sensitive calcium channels and the NMDA receptor function; and finally, at the neuronal non-synaptic level, it hyperpolarizes neurons by modulating potassium channel conductance. Finally, R-PIA, by activating A1R, reduces cathepsin D release and increases the levels of survival proteins AKT and HSP70 as well as the anti-apoptotic response.

In previous studies, we demonstrated the neuroprotective effect of R-PIA in the PILO model [43,44]. In the present study, we demonstrated intense activation of caspases-1 and -3 during the early phase of the SE induced by PILO, followed by a substantial reduction in the activity of these enzymes during the latent phase (7 days). Increased cathepsin D levels in PILO-treated rats were significantly reduced in R-PIA-treated animals 7 days after SE onset. Concomitantly, an essential upregulation of HSP-70 and AKT was observed in the R-PIA+PILO group compared to the PILO group. These data suggest that the neuroprotection effect caused by R-PIA in the PILO model [43,44] involves downregulation of caspase-1, -3 activity and cathepsin D, and upregulation of the survival factors HSP70 and AKT as cellular mechanisms. Some mechanisms related to neuroprotection resulting from A1 receptor activation are shown in Figure 6.

## 4. Materials and Methods

### 4.1. Animals

Adult male Wistar rats, weighing approximately 250 g, were housed under standard controlled conditions (12/12-h light/dark cycle; 20–22 °C; 40–60% humidity) with food and water offered ad libitum. All animal procedures were conducted per national and international legislation (Guidelines of the National Council for the Control of Animal Experimentation; NIH Guide for Care and Use of Laboratory Animals) and were approved by the Ethical Committee of our University (CEUA 1770/2006). Efforts were made to minimize the number of animals used and to avoid their suffering.

### 4.2. Pilocarpine and R-PIA Protocols

Seizures were induced by pilocarpine, as reported previously [39]. Briefly, rats were injected with pilocarpine hydrochloride (360 mg/kg, Merck, Darmstadt, Germany), administered intraperitoneally (i.p.) 20 min after the subcutaneous (s.c.) injection of methyl-scopolamine. Methylscopolamine (1 mg/kg, s.c., Merck, Darmstadt, Germany) was used to minimize peripheral consequences of pilocarpine such as diarrhea, piloerection, and olfactory and gustatory automatisms associated with salivation, eye blinking, vibrissae twitching, and yawning that usually start 5–10 min after pilocarpine injection [6]. Pretreatment with the adenosinergic A1 receptor agonist R-PIA (Merck, Darmstadt, Germany) was performed as described previously [43]. Briefly, R-PIA was dissolved in dimethylsulfoxide (DMSO) (Merck, Darmstadt, Germany) and saline at a ratio of 1:5 (*v*/*v*), and a dose of 0.025 mg/kg was given (i.p.) 15 min before pilocarpine or saline. The adenosine receptor antagonist 8-(p-sulfophenyl) theophylline (8pSPT) dissolved in DMSO/saline, which does not cross the blood–brain barrier, was used to reduce the peripheral effects of adenosine, and was given (i.p.) at 1.5 mg/kg, at the same time as R-PIA, but using different syringes [48]. The injection volume was 1 mL/kg of body weight. Control animals were given an equivalent volume of vehicle or saline at the respective times. A total of 56 animals were needed for the study groups.

### 4.3. Groups

Following treatments, rats were randomly divided into four groups: Saline: rats injected with saline following DMSO/saline; PILO: rats treated with pilocarpine following DMSO/saline injection; R-PIA+SALINE: animals treated with saline following co-administration of R-PIA and 8pSPT; and R-PIA+PILO: animals treated with pilocarpine following R-PIA+8pSPT injection. All rats treated with pilocarpine exhibited the sequence of behavior changes described by Turski et al. [6] and Leite et al. [7]

### 4.4. Caspase Assay

Caspase assays were performed using a method described by Thornberry et al. [49] and modified by Belizário et al. [65] Briefly, rats from all groups (*N* = 5/group) were euthanized under deep anesthesia at 1 h 30 min and 7 days following SE. Hippocampi were dissected at 4 °C and added to 20 mM HEPES buffer (pH 7.4) that contained 2 mM EDTA, 0.1% CHAPS, 10% sucrose, 0.1% PMSF, 0.1% benzamidine, 0.1% antipain, 0.1% TLCK, 0.1% chymostatin, and 0.1% pepstatin (5 μL homogenization buffer/mg tissue). Homogenates were obtained by mechanical disruption in a Dounce homogenizer, followed by three cycles of freezing in dry-ice and thawing at 4 °C. Samples were centrifuged at 12,000× *g* for 40 min at 4 °C to remove cellular debris. Total proteins were determined in the supernatants using the Bio-Rad Protein Assay (Bio-Rad, Hercules, CA, USA). Homogenates (100 μg/protein) were incubated at 37 °C with the fluorogenic substrates and peptide inhibitors, respectively, as follows: caspase-1, Ac-WEHD-AMC (4 μM) and Ac-WEHD-CHO (1 μM); caspase-3, Ac-DEVD-AMC (4 μM) and Ac-DEVD-CHO (1 μM). The enzymatic reaction was performed in duplicate by incubating samples (15 min at 37 °C) with 100 mM HEPES buffer, pH 7.5, 10 mM dithiothreitol (DTT), in a total volume of 1.5 mL. Samples were transferred to cuvettes, and 10–50 µM of the substrate was added. The activity was measured continuously over 90 min in a GENius Tecan (Austria GMBH) fluorometer using an excitation wavelength of 360 nm and an emission wavelength of 465 nm. The fluorescence of the cleaved substrate was compared with that observed in the presence of a 100 × concentration of a specific caspase inhibitor. The proteolytic reaction velocity (RFU/min) was obtained using the following formula: [FU1 − FU0/ T1 − T0] × 60, which represents the difference in fluorescence units (FU) between time initial (TO) and time final (T1) of the reaction.

### 4.5. Western Blot

The proteins AKT, HSP-70, and cathepsin D were analyzed using Western Blot of hippocampal samples of rats of all studied groups (*N* = 4/group), euthanized 7 days following SE. Hippocampi were homogenized with lysis buffer containing 50 mM Tris-HCl (pH 8.0), 150 mM NaCl, 0.1% SDS, 1% Triton X-100, and a 1% cocktail of protease inhibitors (Merck, Damstadt, Germany). The samples were sonicated in an ice bath. The suspensions were centrifuged at 12,000× *g* for 40 min at 4 °C. The protein content of supernatants was determined using the Bradford method [47]. Protein samples in lysis buffer were mixed with the same volume of Laemmli sample buffer (Bio-Rad, Hercules, CA, USA) and boiled for 10 min, 40 µg of each protein sample was loaded in each well and separated by 12% SDS-PAGE in Mini Protean cells (Bio-Rad, Hercules, CA, USA), and then proteins were electroblotted onto PVDF membranes (Millipore, Burlington, MA, USA) using wet blotting (200 mA for 3 h 30 min). The membranes were washed three times in TBST (50 mM Tris, pH 7.5, 150 mM NaCl, 0.1% Tween 20) and then blocked in TBS (50 mM Tris, pH 7.5, 150 mM NaCl) for 1 h at room temperature. The membranes were probed with the primary antibody for AKT (1:2000), HSP-70 (1:3000), cathepsin D (1:75), or β-actin-peroxidase (1:3000, monoclonal) in blocking buffer, and incubated overnight at 4 °C. The membranes were then washed three times in TBST. They were incubated over 2 h in blocking solution containing each the following HRP-conjugated secondary antibodies: AKT (1:500, anti-rabbit), HSP 70 (1:5000, anti-mouse), and cathepsin D (1:500, anti-rabbit). All antibodies were obtained from Merck (Darmstadt, Germany). Then, the membranes were washed three times in TBST, and bands containing hippocampal proteins were visualized using an ECL detection system Pierce Protein Research Products (Thermo Fisher Scientific, Waltham, MA, USA) on photographic film. 

### 4.6. Statistical Analysis

Data are displayed as means ± Standard Deviation (SD). Comparisons between the groups were made with the Kruskal–Wallis test and post hoc Wilcoxon Signed Rank Test since all proteins presented non-parametric distribution based on Shapiro–Wilk and Levene tests. The Benjamini–Hochberg procedure was applied to adjust all p values. Statistics were carried out in RStudio version 1.1.463 with R version 3.5.2, and the libraries stats and car. A value of *p* < 0.05 was considered statistically significant.

## 5. Conclusions

R-PIA administered previously to PILO-induced SE strongly and significantly increased the activity of caspase-1 and -3 in the first hour following its application. However, it significantly reduced their activities 7 days later compared to non-R-PIA-treated rats. Downregulation of cathepsin D by R-PIA with concomitant upregulation of survival factors HSP-70 and AKT suggest an essential role for adenosine A1 receptor in the neuroprotection following SE.

## Data Availability

The data presented in this study are available in the main text or on request from the corresponding author.
